# Hot Springs on the Overland Route to California

**Published:** 1851-12

**Authors:** G. S. Walker

**Affiliations:** Sacramento, California; and of the Jefferson Medical College


*Hot Springs on the Overland Route to California.* By G. S. Walker, Sacramento, California; and of the Jefferson Medical College. Communicated by Professor Dunglison.

*Philadelphia, Nov.* 23, 1851.

Dr. Dunglison:

*Dear Sir,*—In accordance with your request I transmit you a short account of the boiling springs I visited during the summer months of 1849, and a description of their singular effects upon those of us who drank of the water.

These springs are situated, as well as I can judge, near the 40th parallel of north latitude, and $40\frac{1}{2}°$ of longitude west of Washington city, and are directly upon the California trail leading from the *sink* of Humboldt river to Pyramid Lake—the terminus of Truckie river.

The whole country around seems to be volcanic, and is now a vast desert, utterly and entirely destitute of vegetation, and affording no fresh water to the traveller for a distance of 75 miles. The springs, I may remark, are not those visited by Colonel Fremont. Our little company of thirteen men reached these springs in the forenoon of a hot July day, weary, hungry and thirsty ; having travelled 50 miles without rest, and without water—save a small quantity of a brackish, insipid article, which we had obtained, and carried in our canteens, and which was exhausted long before reaching this place. The springs I did not count, but they may number 200 within a circuit of as many yards. Each spring seems to have perforated the rock, making holes varying in diameter from two or three inches, to the largest which is twenty-five or thirty feet. The rock seems to be a carbonate of lime.

The various springs differ in temperature, some being almost cold, while others are at the temperature of the boiling point.

Some had the nauseous taste of a strong solution of sulphate of magnesia. One or two discharged a blueish-white, thick, mortar-like substance, which I judged from its taste and appearance to be principally a thick solution of carbonate of magnesia. From one of these, and the largest of all, I procured two small bottles-full, for the express purpose of sending the water to you to analyze ; but an accident deprived me of them.

Not the least curious feature of these springs is their ebbing and flowing, which take place at irregular intervals. The flowing is preceded by a loud subterranean noise. Some of them overflow for a few minutes, and fall back three or four feet below the surface, while no one of them flows at all times.

The largest, which was at the temperature of 212°, was almost perfectly transparent; so much so that we could distinctly see the bottom, a distance of some twelve or fifteen feet. This we deemed the least unpleasant to the taste, and of the water we made coffee of which we all drank freely. After leaving the springs, which we did in a couple of hours, we directed our course toward Pyramid Lake, a distance of some twenty-five miles. Some of our party were taken sick in an hour after, and every individual within twenty-four hours. Some were first attacked with vomiting, but more generally it commenced with languor and sickness, and an itching of the orifice of the urethra, followed by severe ardor urinæ, pains in the groin, thighs and testicles. In all the cases there was a constant and inordinate desire to void the urine. In many, a slight hæmorrhage occurred, and in others a discharge of a thick, glairish, pellucid matter from the urethra. Those who were first attacked, suffered the most, but recovered most quickly—the affection only lasting a few hours; while those who escaped until the next day, suffered much longer but not so severely. Plenty of fresh water, and a few doses of Dover's powder combined with a small portion of camphor seemed to afford relief. All recovered within two days; and our sufferings would soon have been forgotten, had it not been for the occasional jests of some of the party about the gonorrhœa we had caught upon the plains. I may add, that all the animals—horses, mules and cattle—that drank tolerably freely of the water, had many of the same symptoms; and some died, doubtless—it seemed to me—from the effect of the water.

Yours, very truly,

G. S. Walker.

